# Blood Pressure and Salivary Cotinine Levels in Young Adults Using Heated Tobacco Products: A Case–Control Study in Poland

**DOI:** 10.3390/healthcare14050600

**Published:** 2026-02-27

**Authors:** Małgorzata Znyk, Hanna Jerczyńska, Leokadia Bąk-Romaniszyn, Dorota Kaleta

**Affiliations:** 1 Department of Population Studies, Medical University of Lodz, Zeligowskiego 7/9, 90-752 Lodz, Poland; dorota.kaleta@umed.lodz.pl; 2 Research Laboratory CoreLab, Medical University of Lodz, Mazowiecka 6/8, 92-215 Lodz, Poland; hanna.jerczynska@umed.lodz.pl; 3 Department of Epidemiology and Public Health, Medical University of Lodz, Zeligowskiego 7/9, 90-752 Lodz, Poland; leokadia.bak-romaniszyn@umed.lodz.pl

**Keywords:** blood pressure, cotinine, heated tobacco product, IQOS, pulse, salivary

## Abstract

**Background/Objectives**: Heated tobacco products (HTPs) are a gateway to nicotine addiction for non-smokers, especially young people. The short- and long-term health effects of using heated tobacco products are not yet fully understood. The study aimed to assess the effect of heated tobacco use on blood pressure and heart rate in young, healthy individuals aged 18–30. The study also assessed exposure to tobacco smoke by measuring salivary cotinine concentration. **Methods**: The case–control study was conducted in 2022–2025 among 200 healthy individuals aged 18–30 years: 70 I-Quit-Ordinary-Smoking users (IQOS), 65 daily traditional cigarette smokers (DS), and 65 non-smokers (NS). The research tool was a questionnaire containing information on the use of tobacco products. The participants completed a questionnaire and then underwent blood pressure measurements, anthropometric measurements, and saliva collection for cotinine levels. **Results**: The average age of initiation of IQOS use was 18.5 years, and smoking had continued for an average of 2.3 years. The average age of initiation of smoking traditional cigarettes was 16.3 years, and smoking had continued for 4.4 years. There were no statistically significant differences in median values between systolic blood pressure (SBP) and diastolic blood pressure (DBP) between the IQOS, DS, and NS groups (*p* > 0.05). High SBP values ≥ 140 mm Hg were observed in 10% of the IQOS users, 18.5% of the daily smokers of conventional cigarettes, and 12.3% of the non-smokers. High DBP values ≥ 90 mm Hg were observed in 11.4% of IQOS, 7.7% of DS, and 7.7% of NS. The cigarette smokers demonstrated significantly higher median cotinine levels compared to the IQOS users and non-smokers: 153.7 vs. 64.3 vs. 0.5 ng/mL (*p* < 0.01). Salivary cotinine levels were positively correlated (ρ = 0.38; *p* < 0.01) with the daily number of heated tobacco sticks among IQOS users (weak correlation), as well as among DS (ρ = 0.42; *p* < 0.01) with a higher daily number of cigarettes (moderate correlation). **Conclusions**: Long-term studies are needed to determine the health effects of heated tobacco products among young people in Poland. Furthermore, the potential impact of HTP aerosols on passive smokers should be examined. Further studies should consider the use of salivary cotinine as a biomarker.

## 1. Introduction

Conventional tobacco smoking has declined over the past decade due to the introduction of new smoking products worldwide [1]. One of these products, alongside e-cigarettes, is a heated tobacco product (HTP).

Heated tobacco products are new devices that heat glycerin sticks and tobacco at low temperatures, up to about 350 °C, without burning the tobacco [2,3,4]. For comparison, in conventional cigarettes, tobacco is burned above 600 °C [1]. E-cigarettes, on the other hand, heat liquids that typically contain nicotine from tobacco, as well as flavorings and other chemical ingredients [2,3].

Both e-cigarettes and HTPs release nicotine in the form of an aerosol without traditional combustion, which is then absorbed via the human respiratory tract. E-cigarettes vaporize a liquid containing flavorings, propylene glycol and/or glycerol, and optionally nicotine. HTPs, on the other hand, heat-processed tobacco leaves, and vaporize residual water along with nicotine [5]. These devices produce aerosols containing toxic chemicals and nicotine when the tobacco-containing device is activated or when the tobacco is heated. These harmful substances are inhaled by HTP users [6]. The aerosol generated during HTP use has a chemical composition similar to traditional cigarettes, including levels of nicotine, glycerol, acetol, propylene glycol, carbonyls, N-nitrosoanabasine, aldehydes, ammonia, volatile organic compounds, carbon monoxide, and polycyclic aromatic hydrocarbons [7]. Tobacco in heated tobacco products usually comes in the form of plugs, sticks, or capsules, which are inserted into a holder and heated by an electronic element [8].

HTP was initially introduced in 2014 in Japan and Italy as a pilot project and is currently sold in over 40 countries worldwide [9], including 21 European countries [10,11]. In Poland, these products appeared in 2017 [12]. According to the latest nationwide survey conducted in Poland in 2022, 4% of adults use heated tobacco [13,14].

Currently, there are three types of heated tobacco products available in Poland, i.e., glo (by BAT- British American Tobacco), I-Quit-Ordinary-Smoking (IQOS) (PMI- Philip Morris International), and Ploom TECH (JTI- Japan Tobacco International) [12,15,16].

Glo is a heating device in which the tobacco rod is heated from the periphery. Each heating session lasts 3 min, reaching operating temperature in approximately 30–40 s [12]. In IQOS, the tobacco stick is placed on a heating blade, and a session can last 4 to 6 min [17]. Ploom uses “Heat Flow” technology, in which the tobacco is heated within a closed chamber, ensuring even airflow around the stick. The tobacco sticks do not have direct contact with the heating element [18].

HTP devices are available for purchase both online and in retail outlets, often displayed in prominent locations likely to be seen by young people. The latest version of IQOS delivers a similar amount of nicotine and is as effective at reducing cravings as regular cigarettes [19].

Heated tobacco products are designed by manufacturers to deliver nicotine in concentrations and quantities similar to those found in cigarettes, with the aim of encouraging current smokers to start and continue using heated tobacco. Nicotine is not only addictive but also toxic for the human body, disrupting the circulatory and respiratory systems, among others. It can also have a lasting negative impact on brain development during adolescence [19].

The attractiveness of these products for teenagers and young people is increased by their availability in various flavors (menthol, fruit) [19]. The variety of flavors also makes them attractive to those who want to gain new smoking experience.

Tobacco companies heavily promote HTP as a less harmful alternative to traditional cigarettes [20] that “reduces harm” [21]. These products are advertised as stylish, modern, and based on a simple design [11]. The main marketing narrative for heated tobacco products is constructed around their lower harmfulness and reduced risk as compared to traditional cigarettes.

For conventional tobacco smokers, HTP can be an alternative option that helps reduce exposure to potentially hazardous and dangerous ingredients. Furthermore, nonsmokers who use HTP may increase their exposure to certain substances and develop dependence, which can increase the likelihood of tobacco-related diseases [22]. Understanding the attractiveness of these products for both smokers seeking an alternative to traditional cigarettes and non-smokers, particularly young people, is important for assessing their public health potential [23].

In light of reports on highly effective consumption of heated tobacco products, special attention should be paid to the potential effects of HTP nicotine consumption in adolescents, pregnant women, and patients with comorbidities [24]. Currently, several independent studies on HTP have been conducted, demonstrating its impact on health [3,25,26]. As a result, these devices are sold without any health warning labels [2]. Public health is now facing a new challenge of introducing large graphic health warning labels for these products, the same as those required for other tobacco products.

The World Health Organization (WHO) does not recommend heated tobacco products for current smokers as a nicotine replacement therapy [27]. These products are a gateway to nicotine addiction for non-smokers, especially young people. The WHO considers these products as harmful to health [28], as is the case with e-cigarettes [24].

Smoking traditional cigarettes is known to increase heart rate, blood pressure, and arterial stiffness parameters [29]. A few studies have also shown that heated tobacco products increase blood pressure [17,20,30,31,32,33] and arterial stiffness [5,20,32,33], leading to a higher risk of hypertension [31]. Increased pulse wave velocity has also been demonstrated in HTP users [20]. Furthermore, arterial stiffness parameters have been found to increase after HTP consumption [33,34]. Additionally, like traditional cigarettes, HTPs have been shown to increase the risk of nicotine-related cardiovascular stress and thus a higher chance of cardiovascular disease [20].

The short- and long-term health effects of using heated tobacco products are not yet fully understood. Research in this area is ongoing worldwide. Our study was designed to fill the research gap in this area.

The study aimed to assess the effect of heated tobacco use on blood pressure and heart rate in young, healthy individuals aged 18–30. The obtained results were compared with those of smokers and non-smokers. The study also assessed exposure to tobacco smoke by assessing salivary cotinine concentration.

## 2. Materials and Methods

### 2.1. Study Design

A case–control study covering the years 2022–2025 included 200 healthy adults aged 18–30 years, recruited from universities and post-secondary schools in Lodz, Poland. Invitations to participate were posted on university websites and social media. Information about the study was also available at IQOS sales points and on university premises (University of Lodz, Medical University of Lodz, and Lodz University of Technology).

### 2.2. The Study Population

Based on self-assessment of the smoking status, the study participants were divided into three groups: I. IQOS users (*n* = 70), II. daily traditional cigarette smokers (*n* = 65), and III. non-smokers (*n* = 65).

The study invitations provided detailed information about the recruited groups, including the criteria for participation.

Individuals who volunteered for the study self-reported their group membership, which was subsequently confirmed by the researcher through an oral interview.

The participants completed a questionnaire and then underwent blood pressure measurements, anthropometric measurements, and saliva sampling for cotinine levels. The questionnaire included sections on smoking and the use of heated tobacco, which were completed by participants according to their group affiliation.

Those in the groups of IQOS users and the daily traditional cigarette smokers had only one smoking habit.

Inclusion criteria for IQOS users: smoking only IQOS, and smoking at least five sticks of IQOS per day for at least six months before study recruitment, 18–30 years of age. Inclusion criteria for daily traditional cigarette smokers (DS): smoking five or more cigarettes per day for at least a year, age 18–30 years. Inclusion criteria for non-smokers (NS): participants who never smoked or used any tobacco substitutes, aged between 18 and 30 years. Exclusion criteria: co-existing diseases, history of surgery within three months preceding the examination, alcohol addiction or regular use of psychoactive substances. The study recruited individuals using the IQOS brand, which was first introduced to the Polish market in 2017. These products have been available on the market for the longest time and are most frequently used by young people.

Two hundred participants took part in the study and were provided with a paper questionnaire, all of which were completed and returned. All participants had their blood pressure and heart rate measured, anthropometric measurements taken, and a saliva sample collected for analysis.

Detailed information on the study methodology, inclusion, and exclusion criteria can be found elsewhere [35]. This article is part of the research project “Heated tobacco use and the health status of young people in Poland” (grant number 2021/41/N/NZ7/00020).

The study was conducted in accordance with the ethical principles of the Declaration of Helsinki. Prior to commencement of the study, a positive opinion was obtained from the Bioethics Committee at the Medical University of Lodz (ref. no. RNN/290/21/KE, 14 December 2021). Having been informed about the purpose and course of the study, all the subjects gave their written consent to participate in it.

### 2.3. Data Collection Methods

#### 2.3.1. Questionnaire

For the purposes of this study, a standardized questionnaire was adapted from the Global Adult Tobacco Survey (GATS) [36,37]. Selected questions from the questionnaire were used for this article.

The questionnaire included data on socio-demographic characteristics (age, gender, education level, place of residence, marital status, family history of hypertension), smoking status (IQOS or tobacco smoking or not smoking), and passive exposure to nicotine smoke. The questionnaire also included questions on participants’ health status.

The users of IQOS were asked about the circumstances and age at which they used IQOS for the first time, frequency of use during the day, and the choice of a heated tobacco stick.

Traditional cigarette smokers were asked about the age at which they started smoking traditional cigarettes, number of years they had been smoking traditional cigarettes, and type and quantity of cigarettes they smoked.

Exposure to passive smoking was assessed based on the following questions: “Are you exposed to passive smoking at home or work?” and “Have you been in rooms where someone smokes?”

#### 2.3.2. Anthropometric Measurements

Anthropometric measurements included height (m), weight (kg), waist circumference (cm), and hip circumference (cm). The participants were barefoot and lightly clothed. Body weight was measured to the nearest 0.1 kg using a digital scale (Manta Multimedia MM441, Warsaw, Poland), and height was measured to the nearest 0.1 cm using a wall-mounted stadiometer (Seca 216, Hamburg, Germany).

Based on the anthropometric measurements, body mass index (BMI) was calculated. Based on the height (m) and weight (kg), the BMI (kg/m^2^) for each respondent was calculated according to the formula: weight (kg) divided by height squared (m^2^). The study subjects were divided into four groups according to BMI: <18.5 kg/m^2^ underweight, 18.5–24.9 kg/m^2^ normal, 25.0–29.9 kg/m^2^ overweight, and ≥30 kg/m^2^ obese [38].

Hip circumference was measured at the widest point over the greater trochanter, and waist circumference was measured midway between the lowest palpable rib and the iliac crest using a flexible anthropometric tape (GIMA, Gessate, Italy). The measurements were performed in duplicate and averaged.

The WHR (waist–hip ratio) was calculated by dividing the waist circumference (cm) by the hip circumference (cm); values >80 cm in women and >94 cm in men indicated abdominal obesity [39].

#### 2.3.3. Blood Pressure Measurement

Blood pressure was measured to the nearest 1 mmHg using an electronic sphygmomanometer UA-611/UA-651 using the oscillometric method (A&D Medical, Kitamoto, Japan). Pulse rate and peripheral blood pressure (systolic and diastolic blood pressure) were measured using a common cuff. The measurement was performed with the subject seated in a quiet room. Five minutes before the blood pressure measurement began, the cuff was placed at the heart level. The measurement was performed according to the ESH/ESC recommendations [40]. The patients were instructed to avoid physical exercise and stimulants (caffeine, tobacco, alcohol) for at least 30 min before blood pressure measurement. Study participants also did not take any medications. Office blood pressure results: non-elevated BP < 120/80 mmHg; elevated BP 120/80–140/90 mmHg, and hypertension ≥ 140/90 mmHg [41].

#### 2.3.4. Determination of Cotinine in Saliva

To verify information on active and passive exposure to tobacco smoke, salivary cotinine analysis was performed at the Corelab Laboratory of the Medical University of Lodz. Cotinine, a biomarker of tobacco smoke exposure and the main metabolite of nicotine, is most commonly used due to its relatively long half-life and high specificity for tobacco smoke. Cotinine is eliminated from the body within several dozen hours, which allows for detecting its presence in the body following passive exposure to tobacco smoke and assessing its quantity even within a few days [42]. The subjects did not consume food within 60 min before testing and did not consume alcohol within 12 h before testing. To minimize acidic or sugary foods, which could lower the sample’s pH, affect bacterial growth, and impair test performance, the subjects thoroughly rinsed their mouths with water for ten minutes before sampling. Approximately 2.0 mL of saliva was required to perform the assay. Samples were collected using passive, unstimulated salivation into disposable sterile tubes. The saliva was passed through a SalivaBio collection device (SCA) into a polypropylene vial. Download protocols/methods are available online at www.salimetrics.com [43].

The obtained samples were stored in a refrigerator within 30 min of collection and frozen at −20 °C or below within four hours of collection. They were then transported to the CoreLab University Research Laboratory in Lodz for cotinine determination. Samples may be stored at −20 °C for up to six months.

Salivary cotinine concentrations were determined using a commercially available ELISA kit (Salimetrics, LLC, State College, PA, USA). Before analysis, the samples were thawed and centrifuged at 1500× *g* for 15 min to remove any debris. The assay was performed according to the manufacturer’s instructions. Briefly, standards, controls, and samples were assayed in duplicate on microplates precoated with cotinine antibodies, where cotinine in the specimens competed with cotinine conjugated to horseradish peroxidase (HRP) for antibody binding sites. After washing with a Stat-Matic Plate Washer II (Sigma-Aldrich, Merck KGaA, Darmstadt, Germany) to remove unbound components, tetramethylbenzidine (TMB) substrate was added to initiate a colorimetric reaction. Color development was stopped with an acid solution, and absorbance was measured at 450 nm using a VICTOR™ X4 Multilabel Plate Reader (PerkinElmer, Shelton, CT, USA). Concentrations were calculated by interpolation from a four-parameter logistic (4PL) standard curve, and data analysis was performed with WorkOut 2.5 Software.

The cut-off concentration of cotinine in saliva that allows for distinguishing between a smoker and a non-smoker is 10 ng/mL [44].

### 2.4. Statistical Analysis

Data analysis was performed using Statistica 13.3 (StatSoft, Krakow, Poland, licensed by the Medical University of Lodz). Quantitative variables were analyzed by calculating the median, mean, standard deviation, and minimum and maximum values. Qualitative variables were analyzed by calculating the number and percentage of occurrences of each value. Categorical data were compared using chi-square tests. The Shapiro–Wilk test was used to test for normality of distribution for continuous variables. The nonparametric Kruskal–Wallis test was used for continuous variables that were not normally distributed. If differences were significant, additional post hoc tests were performed to distinguish between groups (Dunn’s test with Bonferroni correction).

Spearman’s rank correlation was used to analyze the correlation between salivary cotinine levels and the number of heated tobacco cigarettes used daily. Spearman’s rank correlation was also used to analyze the correlation between salivary cotinine levels and the number of traditional cigarettes smoked per day. Correlation strength ρ > 0.9 indicates a very strong relationship; ρ = 0.7–0.9 indicates a fairly strong relationship; ρ = 0.4–0.7 indicates a moderate relationship; ρ = 0.2–0.4 indicates a weak relationship; ρ < 0.2 indicates no linear relationship. A negative correlation, marked with a “-,” indicates an increase in one variable and a decrease in the other. A positive correlation reflects an increase or a decrease in both variables combined [45].

Multiple regression models were constructed to examine the effects of several independent variables on a single dependent variable. The R-squared value indicates the goodness of fit of the model to the data, with values close to 1.0 suggesting that nearly all variability in the dependent variable is explained by the included independent variables. The adjusted R-squared in multiple regression accounts for the number of predictors when assessing model fit.

These analyses were performed at a significance level of 0.05; therefore, values of *p* < 0.05 were interpreted as statistically significant.

## 3. Results

### 3.1. Characteristics of the Participants

In total, 200 people were recruited for the clinical case study, including 70 IQOS users (IQOS), 65 daily traditional cigarette smokers (DS), and 65 non-smokers (NS).

The mean ages were 21.2 ± 2.1 years for the IQOS users, 21.4 ± 2.5 years for the traditional cigarette smokers, and 21.0 ± 1.9 years for the non-smokers. A majority of the respondents were women (71.4% of IQOS users, 58.5% of traditional cigarette smokers, and 86.2% of non-smokers). Most of the participants lived in cities with over 200,000 inhabitants (64.3% IQOS, 55.4% DS, and 47.7% NS). Normal body weight was observed in 67.2% of IQOS users, 53.8% of traditional cigarette smokers, and 73.9% of non-smokers. In the IQOS and NS groups, 40% of the respondents had a family history of hypertension, while in the DS group, it was approximately 41.5%.

The average age of initiation of IQOS use was 18.5 years, and smoking had continued for an average of 2.3 years. The average age of initiation of smoking traditional cigarettes was 16.3 years, and smoking had continued for 4.4 years. Detailed data on the sociodemographic characteristics of the participants are included in Table 1.

### 3.2. Anthropometric Measurements

Detailed anthropometric measurements in the three analyzed groups are provided in Table 2. Statistically significant differences (*p* < 0.001) were found in anthropometric measurements (body weight, height, waist circumference, hip circumference, and BMI) between the IQOS, DS, and NS groups.

### 3.3. Passive Exposure to Tobacco Smoke

Approximately 65.7% of the IQOS users indicated they were passively exposed to secondhand smoke, compared to 60% of traditional cigarette smokers and non-smokers, respectively. About 60% of non-smokers and half of daily traditional cigarette smokers did not spend time in rooms where others smoke. In the IQOS group, it was 44.3% of the respondents. Most of the respondents spent less than one hour per day in rooms where others smoked (40% for the IQOS, 20% for the DS, and 26.2% for the NS groups, respectively). Data on passive exposure to tobacco smoke among the respondents are presented in Table 3.

### 3.4. Blood Pressure

The analyzed parameters of systolic blood pressure, diastolic blood pressure, and heart rate were not normally distributed (*p* < 0.05). There were no statistically significant differences in median values between the IQOS, DS, and NS groups (*p* > 0.05). Detailed data are provided in Table 4 and Table S1 in the Supplementary Materials.

High systolic blood pressure (SBP) values ≥ 140 mm Hg were observed in 10% of the IQOS users, 18.5% of the daily smokers of conventional cigarettes, and 12.3% of the non-smokers. High diastolic blood pressure (DBP) values ≥ 90 mm Hg were observed in 11.4% of the IQOS, 7.7% of the DS, and 7.7% of the NS groups. Heart rate values > 100 bpm were observed in 4.3% of the IQOS, 6.2% of DS, and 3.1% of NS (Table 5).

No linear correlation (ρ < 0.02) was found between the studied parameters of blood pressure and pulse and the daily frequency of nicotine-heated tobacco sticks among the IQOS users (*n* = 70) (Table 6). Similarly, no linear correlation was observed between SBP, DBP, and pulse and the number of cigarettes smoked per day among the DS group (*n* = 65) (Table 7), whereas BMI (ρ = 0.28; *p* < 0.05) and WHR (ρ = 0.26; *p* < 0.05) were positively correlated with the daily number of cigarettes smoked. The correlation, however, was weak (ρ = 0.2–0.4) (Table 7).

### 3.5. Salivary Cotinine Levels

**Figure 1:**
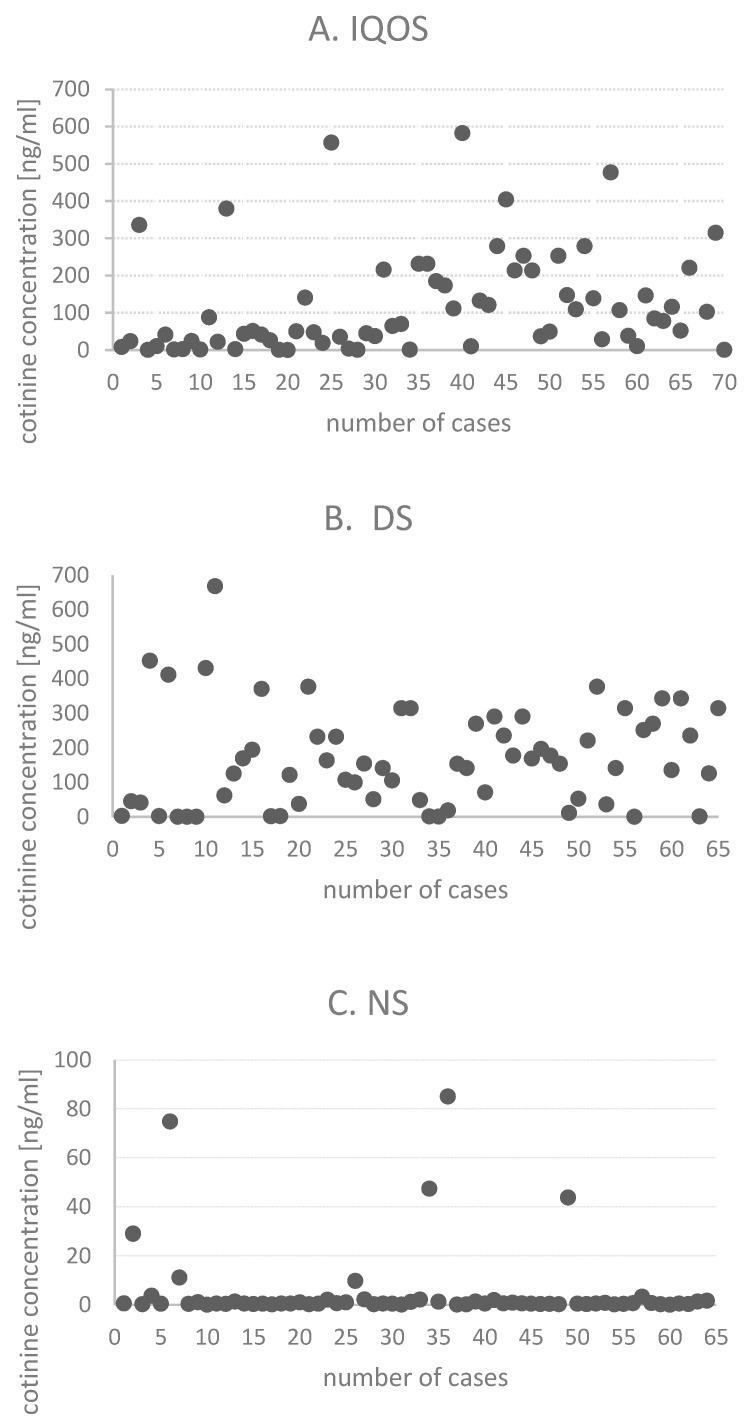
Cotinine concentration levels in the three analyzed groups: (**A**) IQOS (*n* = 70), (**B**) DS (*n* = 65), (**C**) NS (*n* = 65). Note: number of cases—next participant number.

The cigarette smokers demonstrated significantly higher median cotinine levels compared to the IQOS users and non-smokers: 153.7 vs. 64.3 vs. 0.5 ng/mL (*p* < 0.01) (Table 4). Detailed data on salivary cotinine measurements are provided in Table 8 and Figure 1. Post hoc analysis showed significant differences in cotinine levels between the NS and IQOS (5.4 vs. 120.5), NS and DS (5.4 vs. 169.1), and IQOS and DS (120.5 vs. 169.1) groups.

Salivary cotinine levels were positively correlated (ρ = 0.38; *p* < 0.01) with the daily number of heated tobacco sticks among the IQOS users, but the correlation was weak (Table 6). A moderate positive correlation (ρ = 0.42; *p* < 0.01) was found between salivary cotinine levels and the daily number of traditional cigarettes smoked in the DS group (Table 7).

Multiple linear regression models were performed (for dependent variables: SBP, DBP, pulse, salivary cotinine; for independent variables: gender, BMI, age, NS, DS). When conducting multivariate linear regression analysis, smoking-related variables, such as age at smoking initiation and daily cigarette consumption, were included as control variables to improve model specification and robustness.

All the models achieved adjusted R^2^ values below or equal to 0.3, which suggests that they had very small or no effect size. Detailed data are provided in Table 9.

In the model predicting cotinine levels, the adjusted R^2^ was 0.319. Three independent variables were significant, i.e., NS (Beta = −115.396; *p* < 0.001), DS (Beta = 377.193; *p* < 0.01), and male sex (Beta = 42.812; *p* < 0.05) compared to IQOS. BMI (*p* = 0.150) and age (*p* = 0.944) did not have a statistically significant effect. Control variables were significant, i.e., age at smoking initiation (Beta = −26.989; *p* < 0.001), and daily cigarette consumption (Beta = 12.127; *p* < 0.001). This means that they have a real impact on the dependent variable and adequately control for confounding.

## 4. Discussion

Our study is one of the first in Poland to examine the health impact of using heated tobacco products, such as IQOS, in young, healthy people.

In our study, no significant differences in blood pressure and heart rate were observed between the analyzed groups. However, it is important to remember that the study was conducted among young (aged 18–30 years) healthy individuals. IQOS users began using the product around the age of 18 and had been using it for an average of two years. The duration of IQOS use was half that of traditional cigarette smokers.

Other studies also found no differences in blood pressure parameters among current smokers, former smokers, and nonsmokers (*p* > 0.05), except for mean arterial pressure (MAP) and diastolic blood pressure (DBP) in smokers and nonsmokers (*p* < 0.05). Furthermore, smokers tended to have lower systolic blood pressure (SBP) compared with former smokers (*p* < 0.05) [46]. The use of nicotine-containing products (cigarettes) is associated with increased blood pressure and heart rate [17]. Similarly, other studies have shown significant differences in diastolic blood pressure (DBP) and mean arterial pressure (MAP) between smokers and non-smokers [46]. Another study identified a significant increase in the prevalence of hypertension and palpitations in smokers compared to non-smokers (*p* < 0.001). A significant positive correlation was identified between smokers’ systolic blood pressure and duration of smoking (DS) [47]. Smokers had significantly higher SBP and mean arterial pressure (MAP) than non-smokers. However, there were no significant differences in DBP (*p* > 0.05) [47]. In a cross-sectional study on adolescents aged 13–18 years, using data from the Korean National Health and Nutrition Survey (KNHANES), tobacco smoke exposure significantly influenced elevated blood pressure (EBP). The risk of hypertension was more than three times higher in adolescents who were current smokers than in those who did not smoke [48]. A study conducted in the United States (US) among children and adolescents aged 8–19 years suggests that exposure to tobacco smoking is associated with increased blood pressure [49].

Numerous papers have reported an increase in blood pressure after using HTP [10,50]. In the study by Lyytinen et al. [32], it was observed that heart rate and blood pressure transiently increased immediately after HTP inhalation. In the study by Goebel et al. [20], all nicotine-containing products (traditional cigarettes, IQOS, and glo) caused similarly significant increases in blood pressure and arterial stiffness. HTP, like traditional cigarettes, leads to nicotine-related cardiovascular stress, which links these products to increased cardiovascular risk. In the study by Hu et al. [31], exclusive HTP users had a higher risk of hypertension, followed by dual users and exclusive cigarette smokers compared to never-smokers. In the study by Majek et al. [17], a significant increase in blood pressure was observed in groups using nicotine-containing products (e-cigarette users, HTP users, and traditional cigarette smokers). In another study, hemodynamic parameters increased equally after HTP and cigarette consumption [17]. Previous studies have shown a less pronounced increase in blood pressure after HTP consumption [34,51].

Increases in blood pressure and heart rate after using HTP and traditional tobacco may result from the similar levels of nicotine in both products [52].

HTP leads to cardiovascular stress and a sharp increase in nicotine-related arterial stiffness (similar to that caused by traditional cigarettes), which links these products to a higher cardiovascular risk [20].

Nicotine is also a substance toxic to the neurobehavioral and reproductive systems. Animal and in vitro studies indicate that more nicotine may be absorbed from HTP than from cigarette smoke [24].

Cotinine, one of nicotine’s metabolites, is used as a biological marker to assess exposure to tobacco smoke. It has a longer shelf life (almost 20 h, compared to nearly two hours in the case of nicotine), remains in the body for a longer period; therefore it can be used as the most sensitive biomarker for identifying cigarette smokers [53,54].

The hypothesis was that salivary cotinine levels are significantly higher in IQOS users and cigarette smokers than in never-smokers. Our study found that salivary cotinine concentrations were higher in traditional cigarette smokers compared to IQOS users and non-smokers (in descending order). It is important to note that the IQOS users in our study used nicotine-containing cartridges. Reported salivary cotinine levels in IQOS users are slightly lower than those in heavy smokers of conventional cigarettes (150–300 ng/mL). To date, no other studies have been conducted comparing salivary cotinine levels in IQOS users, traditional cigarette smokers, and non-smokers.

Our study also identified a positive and weak correlation between IQOS use and salivary cotinine levels. In this context, increasing IQOS consumption led to higher salivary cotinine levels.

Other studies have shown that e-cigarette and traditional cigarette smokers have similar cotinine levels [55,56]. Also, it has been reported that most daily e-cigarette smokers and co-smokers have high levels of cotinine in their saliva [57,58].

In the study by Hasan et al. [59], cotinine concentrations were higher in cigarette and e-cigarette smokers than in never-smokers (*p* < 0.05). The study by Honarmand et al. [60] showed that participants consuming smokeless tobacco had higher levels of cotinine in their saliva compared to tobacco smokers, and this difference was statistically significant.

The results of other studies have shown a positive and strong correlation between waterpipe smoking and the level of cotinine in saliva [61].

Cotinine levels in the body vary depending on the rate of cotinine metabolism, the rate of its conversion to nicotine, and the daily nicotine intake. Individuals with a lower BMI have a slower rate of cotinine metabolism [62,63]. Cotinine levels are higher in individuals with a lower BMI [62]. Our study did not confirm this relationship.

In our study, the average duration of IQOS use was two years (2.3 ± 1.4), while the average duration of smoking traditional cigarettes was four years (4.4 ± 3.5). Young people at older ages were using IQOS, resulting in shorter exposure to its effects. The regulations currently applied in Poland prohibit the sale of tobacco products, nicotine pouches, electronic cigarettes, or refill containers to persons under 18 years of age. Clear and visible information about the ban on selling them to minors is posted at each retail outlet [64].

Higher salivary cotinine concentrations were observed in smokers who used heated tobacco products more frequently and in smokers who smoked larger amounts of cigarettes. The elevated cotinine levels in IQOS users and cigarette smokers in this study could also be due to exposure to secondhand smoke from family or friends. Almost every third IQOS user and every third smoker of traditional cigarettes in our study was exposed to passive smoking at home or at work.

### Strengths and Weaknesses of the Study

Our study was one of the first to examine salivary cotinine levels in young IQOS users, traditional cigarette smokers, and non-smokers. Furthermore, it was one of the first studies to measure blood pressure in the analyzed groups. The reliability of self-reported tobacco product use was confirmed by measuring salivary cotinine concentration. The participants in our study were mainly students and learners; they were not exposed to harmful workplace factors that could cause negative health effects.

A weakness of the study is its single-point observation. Other limitations are the small sample size as well as reliance on self-reported smoking status from a self-administered questionnaire. Self-declaration by the participants may be biased. The young age of the participants suggests a relatively short period of use of heated tobacco products, which may have influenced the study results. Young individuals may not have yet experienced the effects of IQOS use (such as increased blood pressure). The participants were selected as exclusive IQOS users or exclusive traditional cigarette smokers, but their smoking history may have included other types of tobacco use. The study questionnaire did not contain questions about prior smoking of traditional cigarettes or e-cigarette use.

The reliability of self-reported tobacco use was confirmed by measuring salivary cotinine concentration. The results obtained in the DS and IQOS groups confirm the use of tobacco products.

Since HTPs have been on the market for a short time only, their long-term health effects are not yet known. The study did not show significant differences in blood pressure between groups of young adults; however, it is possible that such effects may emerge with longer use. In fact, the findings are contrary to what might be expected, as smokers exhibit higher blood pressure values than non-smokers, although the literature demonstrates the cardiovascular effects of nicotine. In this regard, future studies would benefit from reassessment of these parameters after a longer follow-up period or in populations with a longer history of tobacco use. This would allow for evaluation of long-term effects.

## 5. Conclusions

Although our study did not show that the use of heated tobacco products increases systolic and diastolic blood pressure, it may serve as a pilot study for the design of similar studies by researchers in Poland and other countries. Further research is needed to determine whether long-term use of heated tobacco products leads to increased blood pressure.

Additionally, the impact of HTP aerosols on passive smokers should be examined. Further studies should consider the use of salivary cotinine as a biomarker. There is a need for regulatory measures to monitor nicotine levels in heated tobacco products.

Preventive campaigns or interventions should be implemented to reduce the use of these products and mitigate their potential health consequences.

## Figures and Tables

**Table 1:** Characteristics of the study participants.

	Total *n* = 200	IQOS *n* = 70	DS *n* = 65	NS *n* = 65
Age (mean ± SD)	21.4 ± 2.5	21.2 ± 2.1	21.4 ± 2.5	21.0 ± 1.9
min-max	18–30	18–30	18–30	19–24
BMI (mean)	23.0	22.7	23.1	22.6
BMI	*n* (%)	*n* (%)	*n* (%)	*n* (%)
underweight (<18.5 kg/m^2^)	17 (8.5)	4 (5.7)	2 (3.1)	11 (16.9)
normal (18.5–24.9 kg/m^2^)	130 (65)	47 (67.2)	35 (53.8)	48 (73.9)
overweight (25.0–29.9 kg/m^2^)	43 (21.5)	15 (21.4)	23 (35.4)	5 (7.7)
obesity (≥30 kg/m^2^)	10 (5)	4 (5.7)	5 (7.7)	1 (1.5)
Gender				
male	56 (28.0)	20 (28.6)	27 (41.5)	9 (13.8)
female	144 (72.0)	50 (71.4)	38 (58.5)	56 (86.2)
Education				
general or vocational secondary schools	83 (41.5)	25 (35.7)	29 (44.6)	29 (44.6)
post-secondary	96 (48)	38 (54.3)	26 (40.1)	32 (49.3)
higher	17 (8.5)	5 (7.1)	9 (13.8)	3 (4.6)
other	4 (2)	2 (2.9)	1 (1.5)	1 (1.5)
Student				
yes	178 (89.0)	62 (88.6)	51 (78.5)	65 (100)
no	22 (11.0)	8 (11.4)	14 (21.5)	-
Marital status				
single	143 (71.5)	43 (61.4)	46 (70.8)	54 (83.1)
married	5 (2.5)	1 (1.5)	3 (4.6)	1 (1.5)
informal, stable relationship	52 (26)	26 (37.1)	16 (24.6)	10 (15.4)
Place of residence				
city up to 200,000 residents	54 (27)	17 (24.3)	22 (33.8)	15 (23.1)
city above 200,000 residents	112 (56)	45 (64.3)	36 (55.4)	31 (47.7)
village	34 (17)	8 (11.4)	7 (10.8)	19 (29.2)
Family history of hypertension				
Yes	81 (40.5)	28 (40)	27 (41.5)	26 (40)
No	119 (59.5)	42 (60)	38 (58.5)	39 (60)
Smoking time (years) (mean ± SD)			4.4 ± 3.5	
Smoking initiation age (mean ± SD)			16.3 ± 1.9	
IQOS usage time (years) (mean ± SD)		2.3 ± 1.4		
IQOS initiation age (mean ± SD)		18.5 ± 2.3		

Note: mean- arithmetic mean; SD—standard deviation; *n*—number of respondents; %—percent; min—minimum; max—maximum; BMI—body mass index; NS—non-smokers; IQOS—IQOS users; DS—daily traditional cigarette smokers.

**Table 2:** Anthropometric measurements in IQOS users, daily smokers, and non-smokers.

	Study Group		Mean	SD	Median	Min	Max	*p*-Value
		*n*						
body weight [kg]	IQOS	70	65.75	13.29	63.70	45	100	<0.001
	DS	65	67.48	14.50	65.00	46	115	
	NS	65	64.97	13.31	63.00	41	95	
height [cm]	IQOS	70	169.84	8.74	169.00	153	194	<0.01
	DS	65	170.52	8.96	170.00	150	197	
	NS	65	169.19	8.57	168.00	156	196	
waist circumference [cm]	IQOS	70	78.07	11.62	76.00	60	113	<0.001
	DS	65	79.08	12.20	77.00	65	128	
	NS	65	77.87	11.85	75.50	59	120	
hip circumference [cm]	IQOS	70	97.71	9.47	97.00	80	123	<0.001
	DS	65	98.98	10.29	98.00	84	141	
	NS	65	97.49	9.71	97.00	66	122	
BMI [kg/m^2^]	IQOS	70	22.66	3.52	22.08	17.26	37.18	<0.001
	DS	65	23.07	3.94	22.28	17.90	39.79	
	NS	65	22.56	3.54	22.04	16.42	33.20	
WHR	IQOS	70	0.80	0.09	0.79	0.65	0.97	0.127
	DS	65	0.80	0.09	0.79	0.65	1.04	
	NS	65	0.80	0.10	0.79	0.61	1.42	

Note: Kruskal–Wallis test.

**Table 3:** Passive exposure to tobacco smoke in the analyzed groups.

Variables	Total *n* = 200 (%)	IQOS *n* = 70 (%)	DS *n* = 65 (%)	NS *n* = 65 (%)	*p*-Value
Exposure to secondhand smoke					
only at home	23 (11.5)	11 (15.7)	6 (9.2)	6 (9.2)	*p* < 0.01
only at work	16 (8)	8 (11.4)	5 (7.7)	3 (4.6)	
at home and at work	22 (11)	4 (5.7)	13 (20)	5 (7.7)	
in other situations	63 (31.5)	23 (32.9)	15 (23.1)	25 (38.5)	
No	76 (38)	24 (34.3)	26 (40)	26 (40)	
Being in rooms where someone smokes tobacco					
I don’t stay in such rooms at all	103 (51.5)	31 (44.3)	33 (50.8)	39 (60)	0.36
less than 1 h during the day	58 (29)	28 (40)	13 (20)	17 (26.2)	
from 1 h to 5 h during the day	25 (12.5)	9 (12.9)	13 (20)	3 (4.6)	
5 to 8 h a day	9 (4.5)	1 (1.4)	4 (6.2)	4 (6.2)	
more than 8 h a day	5 (2.5)	1 (1.4)	2 (3.0)	2 (3.0)	

Note: chi-squared test.

**Table 4:** Comparison of Blood Pressure and Cotinine Medians Across Three Groups (IQOS, DS, NS).

	Median				
	Total (*n* = 200)	IQOS (*n* = 70)	DS (*n* = 65)	NS (*n* = 65)	*p*-Value
systolic blood pressure [mmHg]	123.5	124	123	123	0.10
diastolic blood pressure [mmHg]	77	77	77	77	0.13
pulse [beats per minute-bpm]	75	74	75	74	0.17
cotinine in saliva [ng/mL]	37.6	64.3	153.7	0.5	<0.01

Note: Kruskal–Wallis test.

**Table 5:** Blood pressure and pulse measurement results in the analyzed groups.

Parameter	Range	IQOS *n* = 70 (%)	DS *n* = 65 (%)	NS *n* = 65 (%)	*p*-Value
systolic blood pressure [mmHg]	<120	26 (37.1)	18 (27.7)	33 (50.8)	*p* < 0.05
	120–140	37 (52.9)	35 (53.8)	24 (36.9)	
	≥140	7 (10.0)	12 (18.5)	8 (12.3)	
diastolic pressure [mmHg]	<80	41 (58.6)	40 (61.5)	41 (63.1)	0.80
	80–90	21 (30.0)	20 (30.8)	19 (29.2)	
	≥90	8 (11.4)	5 (7.7)	5 (7.7)	
pulse [beats per minute]	<60	7 (10.0)	6 (9.2)	6 (9.2)	0.76
	60–100	60 (85.7)	55 (84.6)	57 (87.7)	
	≥100	3 (4.3)	4 (6.2)	2 (3.1)	

Note: Kruskal–Wallis test.

**Table 6:** Correlation between the Blood Pressure and Cotinine and the daily frequency of using nicotine-heated tobacco sticks among IQOS users (*n* = 70).

	ρ (rho)	*p*-Value
SBP & daily frequency of using nicotine-heated tobacco sticks	−0.13	0.2979
DBP & daily frequency of using nicotine-heated tobacco sticks	0.07	0.5286
pulse & daily frequency of using nicotine-heated tobacco sticks	0.14	0.2496
BMI & daily frequency of using nicotine-heated tobacco sticks	0.12	0.3070
WHR & daily frequency of using nicotine-heated tobacco sticks	−0.03	0.8093
cotinine in saliva & daily frequency of using nicotine-heated tobacco sticks	0.38	*p* < 0.01

Note: systolic blood pressure—SBP; diastolic blood pressure—DBP; body mass index—BMI; waist–hip ratio—WHR; the Spearman rank correlation coefficient—ρ (rho); significant values (*p* < 0.01).

**Table 7:** Correlation between the Blood Pressure, cotinine, and the number of cigarettes smoked per day (*n* = 65).

	ρ (rho)	*p*-Value
SBP & number of cigarettes smoked per day	−0.03	0.8282
DBP & number of cigarettes smoked per day	−0.06	0.6154
pulse & number of cigarettes smoked per day	−0.01	0.9536
BMI & number of cigarettes smoked per day	0.28	*p* < 0.05
WHR & number of cigarettes smoked per day	0.26	*p* < 0.05
cotinine in saliva & number of cigarettes smoked per day	0.42	*p* < 0.01

Note: systolic blood pressure—SBP; diastolic blood pressure—DBP; body mass index—BMI; waist–hip ratio—WHR; the Spearman rank correlation coefficient—ρ (rho); significant values (*p* < 0.05; *p* < 0.01).

**Table 8:** Cotinine concentration measurement results in the analyzed groups.

	Study Group		Mean	SD	Median	Min	Max	*p*-Value
		*n*						
Cotinine [ng/mL]	IQOS	70	120.5	136.6	64.3	0.09	582.41	<0.01
	DS	65	169.1	143.4	153.7	0.11	667.60	
	NS	65	5.4	16.1	0.5	0.03	85.01	

**Table 9:** Results of multivariate linear regression models. Dependent variable: blood pressure parameters or cotinine in saliva. Control variables: age at smoking initiation, daily cigarette consumption.

	Beta	t	*p*-Value	Adj. R^2^
**Dependent variable: SBP**				0.130
Gender: male	7.556	3.582	<0.001	
BMI	0.761	3.004	<0.01	
Age (years)	−1.047	−2.696	<0.01	
NS	0.288	0.130	0.897	
DS	−25.204	−1.812	0.072	
hypertension in the family	0.755	0.394	0.694	
age at smoking initiation	1.838	2.157	<0.05	
daily cigarette consumption	−0.333	−1.033	0.303	
**Dependent variable: DBP**				0.052
Gender: male	−4.101	−2.837	<0.01	
BMI	0.538	3.100	<0.01	
Age (years)	−0.345	−1.298	0.196	
NS	−1.688	−1.112	0.267	
DS	−1.124	−1.118	0.906	
hypertension in the family	1.788	1.361	0.175	
age at smoking initiation	0.001	0.002	0.999	
daily cigarette consumption	0.062	0.282	0.779	
**Dependent variable: pulse**				0.004
Gender: male	0.735	0.305	0.761	
BMI	0.165	0.572	0.568	
Age (years)	−1.059	−2.389	<0.05	
NS	−1.553	−0.614	0.540	
DS	−7.791	−0.494	0.622	
age at smoking initiation	0.470	0.487	0.627	
daily cigarette consumption	0.101	0.275	0.783	
**Dependent variable: cotinine in saliva**				0.319
Gender: male	42.812	2.225	<0.05	
BMI	−3.339	−1.445	0.150	
Age (years)	−0.249	−0.070	0.944	
NS	−115.396	−5.713	<0.001	
DS	377.193	2.993	<0.01	
age at smoking initiation	−26.989	−3.503	<0.001	
daily cigarette consumption	12.127	4.129	<0.001	

Note: BMI—body mass index; NS—never smokers; DS—daily traditional cigarette smokers; SBP—systolic blood pressure; DBP—diastolic blood pressure; Adj.—adjusted; R^2^—R-squared coefficient of determination; Beta—regression coefficient.

## Data Availability

The data presented in this study are available upon request from the corresponding author, Małgorzata Znyk, in accordance with the confidentiality assured to participants and professional ethical standards.

## Supplementary Materials

The following supporting information can be downloaded at: https://www.mdpi.com/article/10.3390/healthcare14050600/s1, Table S1. Blood pressure and pulse in IQOS users, daily smokers, and non-smokers.

## Abbreviations

The following abbreviations are used in this manuscript:

BAT	British American Tobacco
BMI	Body mass index
bpm	beats per minute
DBP	diastolic blood pressure
DS	Daily traditional cigarette smokers
EBP	elevated blood pressure
GATS	Global Adult Tobacco Survey
HTPs	Heated tobacco products
IQOS	I-Quit-Ordinary-Smoking, IQOS users
JTI	Japan Tobacco International
KNHANES	The Korean National Health and Nutrition Survey
MAP	mean arterial pressure
NS	Non-smokers
PMI	Philip Morris International
R^2^	R square
SBP	systolic blood pressure
US	the United States
WHO	World Health Organization
WHR	Waist–hip ratio
