# Supplementary material for: Blood Pressure and Salivary Cotinine Levels in Young Adults Using Heated Tobacco Products: A Case–Control Study in Poland
Source: Healthcare (Basel). 2026 Feb 27;14(5):600. doi: 10.3390/healthcare14050600 (PMC12984569; doi:10.3390/healthcare14050600)
Supplement: Supplementary file 1 [file healthcare-14-00600-s001.zip › healthcare-4108464-supplementary.pdf]

Table S1. Blood pressure and pulse in IQOS users, daily smokers, and non-smokers.

	Study group	*n*	Mean	SD	Median	Min	Max	*p*- value
systolic blood pressure [mmHg]	IQOS	70	123.99	12.30	124	101	156	0.10
	DS	65	124.20	13.50	123	100	180	
	NS	65	123.72	12.14	123	96	154	
diastolic pressure [mmHg]	IQOS	70	77.28	8.99	77	54	106	0.13
	DS	65	77.12	8.87	77	55	101	
	NS	65	77.28	9.10	77	60	98	
pulse [beats per minute]	IQOS	70	76.60	14.00	74	46	114	0.17
	DS	65	76.72	14.37	75	41	131	
	NS	65	76.41	14.45	74	48	143	

Note: Kruskal-Wallis test.
